# Targeted Determination of Residual Sex Hormones in Cosmetics Using Magnetic Solid-Phase Extraction with Isotope-Labeled Internal Standards by UHPLC-MS/MS

**DOI:** 10.3390/molecules31010090

**Published:** 2025-12-25

**Authors:** Yalei Dong, Shuyan Sun, Yasen Qiao, Chunhui Yu, Haiyan Wang, Lei Sun

**Affiliations:** 1 National Institutes for Food and Drug Control/NMPA Key Laboratory for Research and Evaluation of Cosmetics, Beijing 100050, China; dongyalei@nifdc.org.cn (Y.D.); yan18531864610@163.com (S.S.); qiaoyasen@126.com (Y.Q.); 2 School of Traditional Chinese Pharmacy, NMPA Key Laboratory for Research and Evaluation for Cosmetics, China Pharmaceutical University, Nanjing 211198, China; 3 Pureton Lab Equipment (Shanghai) Co., Ltd., Shanghai 201108, China; 13825165882@139.com

**Keywords:** MSPE, UHPLC-MS/MS, sex hormones, cosmetics, progesterone, safety evaluation

## Abstract

As rapidly developing consumer products, cosmetics confront challenges regarding safety, especially hazardous ingredients, like sex hormones. Prolonged exposure to trace sex hormones in cosmetics can inflict immeasurable damage to human health. To accurately detect the trace amounts of sex hormones in cosmetics, a reliable method was developed and validated using ultra-high performance liquid chromatography–mass spectrometry (UHPLC-MS/MS) with magnetic solid-phase extraction (MSPE) and isotope-labeled internal standards (IL-ISs). The conditions of sample pretreatment, chromatography, and mass parameters were systemically investigated. In the MSPE procedure, the commercial Fe_3_O_4_@HLB magnetic material was employed for sample pretreatment, which was beneficial for operation, as well as sample purification and analyte enrichment. The utilization of IL-ISs compensated for potential matrix effects and losses during sample preparation, thereby improving precision and accuracy. Based on the proposed MSPE technology, UHPLC-MS/MS can address the qualitative and quantitative analysis needs for target analytes in complex cosmetic matrices. At three fortification levels, recoveries were in the range of 71.7–116.2%, with a relative standard deviation (RSD) ranging from 1.6% to 8.3%. Furthermore, based on the method proposed here, a total of 116 batches of cosmetics were analyzed, and trace progestins and estrogens were discovered in 10 samples. The MSPE method, coupled with UHPLC-MS/MS using IL-ISs, was convenient, efficient, and feasible for detecting trace amounts of sex hormones in cosmetics. The method scored 0.66 (out of 1) on the AGREE metric, confirming its green profile. Based on the detected concentrations, a preliminary safety evaluation was performed to assess the potential health risks of residual progesterone in hair loss prevention cosmetics by calculating the margin of safety (MoS).

## 1. Introduction

Exogenous sex steroid hormones, exhibiting a strong affinity for hormone receptors and high disruption potencies in humans, have received increasing attention in recent years [1]. These hormones derive from both natural and synthetic sources. Natural hormones are normally excreted by humans and livestock, involving progestin (P), estrone (E1), estradiol (E2), estriol (E3), and so on. Synthetic hormones are synthetic progestin or estrogen derivatives used in oral contraceptives and hormone replacement therapies, such as norgestrel (NG), ethynyl estradiol (EE2), and the like [2,3]. In recent decades, sex hormones involving P, E1, E2, and E3 were frequently detected at the ng/L level in aquatic environments and the ng/g level in soil [4]. These hormones can enter organisms through three ways, including oral intake, skin contact, and inhalation of atmosphere contaminated with them [5,6]. More recent researches have shown that exposure to these hormones at concentration levels below nanograms per liter (ng/L) is associated with an increased risk of reproductive disorders and carcinogenesis [7,8]. Epidemiological surveys have demonstrated an elevated incidence ratio of breast cancer among women using transdermal estrogen during an extended period [9]. Endogenous steroid hormones contribute to wrinkle reduction and anti-aging by regulating skin physiology, but intentional addition to cosmetics is not permitted [10,11]. Previous survey results show that the illicit use of sex hormones still existed in cosmetics [12,13,14]. Given the serious health risks, it is crucial to determine sex hormones in cosmetics. Nevertheless, cosmetic formulations are complex, containing numerous ingredients, such as surfactants, preservatives, pH adjusters, and so on [15]. The inherent complexity of cosmetics makes it more difficult to purify and analyze. With consideration of these challenges, our objective is to provide a method to detect these trace sex hormones in cosmetics.

To date, the reported analytical methods for sex hormones in cosmetics include high-performance liquid chromatography (HPLC) [16], liquid chromatography–tandem mass spectrometry (LC-MS/MS) [17], HPLC–high-resolution mass spectrometry (HPLC-HRMS) [18], neutral desorption extractive electrospray ionization tandem mass spectrometry [19], matrix-assisted laser desorption ionization time-of-flight mass spectrometry [20], and so on. LC-MS/MS possesses several advantages: efficient separation for LC and the simultaneous identification and detection of multiple compounds at trace level. Even so, LC-MS/MS should be compatible with appropriate sample preparation techniques [21], especially for complicated samples. Cosmetic samples are commonly pretreated by solid-phase extraction (SPE) or liquid–liquid extraction (LLE) [22]. However, these traditional pretreatment technologies are usually time-consuming and multi-step and require large amounts of organic solvents [23]. Consequently, the development of a convenient sample pretreatment method for trace analytes in a complex matrix is essential. 

Magnetic solid-phase extraction (MSPE), an innovative strategy that employs magnetic or magnetizable adsorbents in place of a conventional SPE column, was developed by Safarikova and Safarik [24]. Compared with the traditional SPE, the adsorbents are directly dispersed in sample solutions or suspensions, which largely improve extraction efficiency in MSPE. Accordingly, it has several advantages, including a low volume of organic solvents, high reproducibility, swift sample preparation, and few equipment requirements [25]. Several magnetic adsorbents were synthesized, such as deep eutectic solvent-based magnetic colloidal gel [26], activated carbon with magnetite nanoparticles (AC/Fe_3_O_4_) [27], β-cyclodextrin-decorated magnetic activated carbon [28], and others. However, these synthetic magnetic adsorbents are designed and synthesized in individual laboratories, exhibiting high recognition only for specific chemical substances, which renders them unable to be widely popularized and applied. Herein, the commercial Fe_3_O_4_@HLB magnetic material was chosen to incorporate more chemicals in our experiment, considering the stability of the material and its potential for automation. In addition to necessary sample preparation, LC-MS/MS technology usually encounters the matrix effect (ME), especially MS equipped with an electrospray ionization source (ESI) [29,30]. The use of stable isotope-labeled internal standards (SIL-ISs) is an accepted strategy to reduce ME. Because of the high similarity between the target analyte and SIL-IS, it is most likely to exhibit the same degree of separation behavior and ion suppression/enhancement [31,32]. When the internal standards (ISs) are added to all samples, the response ratio of analyte to internal standard will be free from the matrix. This means that the calibration curve plotting the response ratio between analytes and ISs against the concentration ratio will remain consistent regardless of the type of matrix [30]. Therefore, it is unnecessary to prepare the calibration standard in matrix, which avoids complicated operations.

In the present study, MSPE was used for the extraction and cleaning-up of sex hormones in cosmetics. Based on the MSPE technique, a detective method using UHPLC-MS/MS incorporating IL-ISs was developed to simultaneously determine 16 kinds of sex hormones. The proposed method was applied to cosmetic products, such as cream, lotion, toner, and gel, which displayed high efficiency, enhanced sensitivity, and strong feasibility. To assess the health risks of the detected contaminants, a simplified protocol was employed based on the monitoring data, highlighting potential exposure risks to consumers.

## 2. Results and Discussion

### 2.1. Characterization of Fe_3_O_4_@HLB

The Fe_3_O_4_@HLB material used here is a commercial product, consisting of a Fe_3_O_4_ core with hydrophilic N-vinylpyrrolidone and lipophilic divinylbenzene copolymer attached to its surface [33]. The morphological structure and size distribution of Fe_3_O_4_@HLB were characterized by SEM, as shown in Figure S1. The Fe_3_O_4_@HLB exhibited an approximatively spherical structure. Additional characterization data were from business detection reports. The micron-sized globules were uniformly dispersed, with a mean diameter of 31.4 μm, by a particle size analyzer. The specific surface area and pore size of the adsorbent play a significant role in the adsorption process [34]. The surface area of Fe_3_O_4_@HLB was measured as 648 m^2^/g, and the average pore size was 67 Å. The results show that the adsorbent material was suitable for enrichment of sex hormones. The magnetic properties was another important factor. The saturation magnetic moment was 5 emu/g using a vibrating sample magnetometer. The results indicate that the material can be separated from a solution rapidly with an external magnetic field.

### 2.2. Development of LC-MS/MS Method

To achieve the optimal response, different mobile phase additives, including formic acid, ammonium formate, and ammonium fluoride, were tried to enhance the ionization efficiency of analytes. The results indicate that adding 0.1% of formic acid and 1 mM of ammonium formate in the aqueous phase decreased the sensitivity in negative ESI mode. By contrast, adding ammonium fluoride in the aqueous phase was conducive to the ionization of compounds in both positive and negative ESI modes. Subsequently, a series of concentrations of ammonium fluoride (0, 0.1, 0.3, 0.5, and 1 mM) were optimized. The results are presented in Figure S2. They show that the response intensity for major analytes was higher with 0.1 mM of ammonium fluoride. However, given the reduction in the peak area of estrogenic compounds with an increasing concentration of ammonium fluoride, 0.1 mM of ammonium fluoride paired with acetonitrile was the mobile phase in our study. In addition, column temperature (25, 30, 35, 40, and 45 °C) was also investigated to improve the chromatographic separation. The results in Figure S3 show that most of the analytes exhibited satisfactory separation and response when the column temperature was maintained at 30 °C.

**Figure 1:**
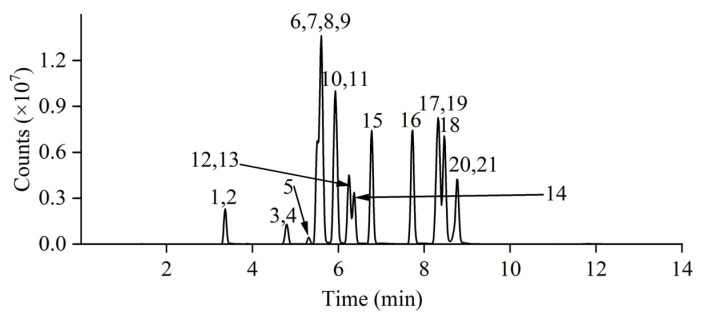
Total ion chromatogram of 16 sex hormones and 5 isotope-labeled internal standards with the peak number consistent with that in Table 1.

To obtain the mass parameters, an individual standard solution was injected into the mass spectrometer with a needle pump. Firstly, the most abundant, [M+H]^+^ or [M−H]^−^, precursor ion was obtained under MS2 scan mode in the *m*/*z* range of 100–1000 Da. Subsequently, two precursor–product ion pair transitions that had maximal signal intensity were identified under product ion scan mode in different collision energies (CEs). Finally, a specific CE was investigated in multiple reaction monitoring (MRM) mode. Information involving the compound name, abbreviation, retention time (RT), and mass parameters is presented in Table 1. The chromatogram is shown in Figure 1, which indicates the satisfactory separation and good response of 16 sex hormones and 5 IS-ILs.

### 2.3. Optimization of Sample Pretreatment

Focusing on cosmetic types with complex formulations [15], prolonged skin contact, and higher risks of illegal additives, this study selected lotion, cream, toner, and gel as representative samples for investigation. Sex hormones present in cosmetics were firstly extracted using acetonitrile, then were purified and enriched through MSPE. In this study, spiked blank samples were prepared: a total of 100 μL of the mixed standard solution of sex hormones, where the concentrations of E3, E3-D3, E2, E2-D5, EE2, HEX, and DIEP were 2 mg/L, and those of the other analytes were 1 mg/L, was added to 0.5 g of blank cosmetic samples to prepare spiked samples, with final spiked concentrations of 0.4 μg/g and 0.2 μg/g, respectively. A simple univariate method was used to optimize the sample pretreatment conditions, and each experiment was carried out in triplicate (n = 3).

Methanol and acetonitrile were investigated to extract sex hormones in cosmetics initially. As shown in Figure S4, higher recoveries were obtained across the cream, gel, lotion, and toner matrix when acetonitrile was used. The sonication time is another factor that influences extraction efficiency. Different extraction times, including 0, 5, 10, 15, 20, 25, and 30 min, were tried. Figure S5 shows that the recoveries of the cream matrix presented a rising trend to some extent when the extraction time stretched from 0 min to 5 min and a sharp descent from 10 min to 30 min. Thus, sex hormones in cosmetics were extracted with acetonitrile for 5 min before MSPE. 

To achieve excellent extraction performance, the type of solid adsorbent, the amount of solid adsorbent, the type of adsorption/desorption solvent, and the time of adsorption/desorption were considered to optimize in MSPE. Given that cream samples have a more complex matrix, they can be selected as representative matrices to investigate the effects of variables in MSPE.

**Figure 2:**
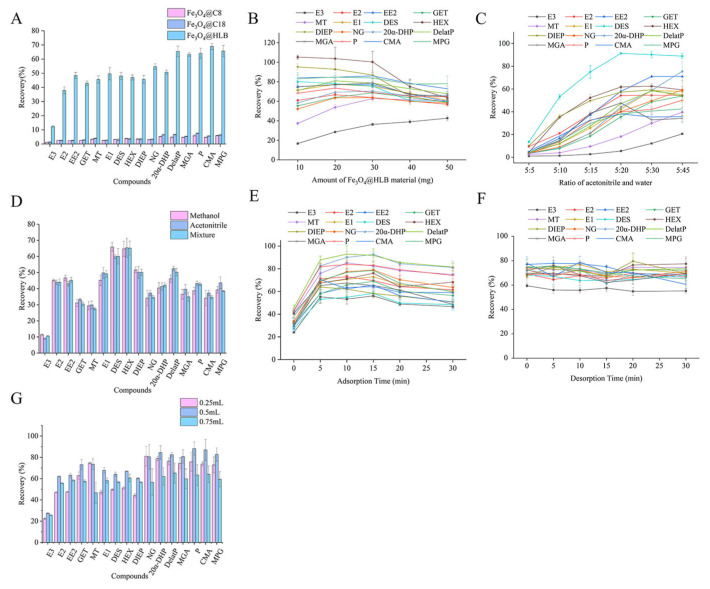
Optimization of MSPE conditions involving the types of adsorbent (**A**), the amount of adsorbent (**B**), the effect of the adsorption solvent (**C**), the effect of the desorption solvent (**D**), the effect of the adsorption time (**E**), the effect of the desorption time (**F**), and the effect of the volume of the desorption solvent (**G**). The error bars represent the standard deviation of three replicate measurements.

The adsorbent material is crucial for MSPE, which enriches target compounds and purifies the cosmetic matrix. Considering the polarity of the target analytes, three types of adsorbents, including Fe_3_O_4_@HLB, Fe_3_O_4_@C18, Fe_3_O_4_@C8, were tried. Fe_3_O_4_@HLB is a kind of hydrophilic–lipophilic reverse-phase extraction material characterized by excellent water wettability. Fe_3_O_4_@C18 and Fe_3_O_4_@C8, coated with silica-based carbons, extract non-polar compounds by hydrophobic interactions. These results indicate that Fe_3_O_4_@HLB had the highest enrichment efficiency (as shown in Figure 2A), suggesting that it was beneficial for hydrogen bonding and π-π interactions with target analytes. Hence, Fe_3_O_4_@HLB was chosen as the adsorbent for the next experiments.

The amount of adsorbent is a significant factor that influences enrichment efficiency. An excessive amount is beneficial for enriching the target compounds but is harmful to the eluent. In contrast, the use of 10, 20, 30, 40, and 50 mg of Fe_3_O_4_@HLB was studied, and the obtained results are described in Figure 2B. The recoveries of major analytes presented an upward tendency within the 10–30 mg range of adsorbent amounts. Beyond 30 mg of adsorbent, the recoveries went down for most of the analytes. Accordingly, 30 mg of adsorbent was chosen for the following experiments.

The adsorption solvent and desorption solvent are essential parameters that affect the interaction between the adsorbent material and target compounds. After extracting with acetonitrile from the cosmetics, a 5 mL extraction solution was collected for further purification. The polarity of the adsorption solvent served as the driving force of adsorption, and thereby, different volumes of pure water (5, 10, 15, 20, 30, and 45 mL) were added to modulate the polarity. Figure 2C shows that the recoveries of the majority of analytes rose to the maximum when 20 mL of pure water was added and then remained stable in defined conditions. However, the recoveries of E3 and EE2, which are regarded as risk compounds in the environment and cosmetics, achieved the maximum when 45 mL of pure water was added. Based on the above, a 5 mL extraction solvent diluted with 45 mL of pure water was employed as the adsorption solvent. An appropriate desorption solvent can weaken the π-π stacking and hydrogen bonding between the adsorbent and target compounds. Three kinds of desorption solvents, including methanol, acetonitrile, and the mixture of methanol and acetonitrile (1:1, *v*/*v*), were respectively evaluated. Figure 2D reveals that the highest recoveries were obtained with acetonitrile among them. In consequence, acetonitrile was set as the optimized desorption solvent.

The adsorption time and desorption time also play an important role in MSPE, as they influence the mass transfer rate of target compounds between the adsorbent and solvent [35]. Due to this, the adsorption times and desorption times at 0, 5, 10, 15, 20, and 30 min were investigated, respectively. As shown in Figure 2E, the recovery increased rapidly as the adsorption time extended from 0 min to 5 min, with a decreased trend thereafter. Figure 2F shows that the recovery for the majority of compounds was higher when the desorption time was 5 min and remained stable with further elongation of the desorption time. As a result, both the adsorption time and desorption time were set to 5 min.

The volume of the desorption solvent is also an essential factor that influences the elution between analytes and adsorbents. A total of 0.25, 0.5, and 0.75 mL of acetonitrile was investigated for the experiment. Figure 2G illustrates higher recovery for the major analytes when the volume of the desorption solvent was 0.5 mL. As a result, 0.5 mL of acetonitrile was selected as the optimal desorption solvent volume.

### 2.4. Method Evaluation

Based on the optimized conditions obtained from the aforementioned pretreatment process, the method was evaluated in terms of linearity, limit of detection (LOD) and limit of quantitation (LOQ), recovery, and intra-day and inter-day precision.

A stable isotope-labeled internal standard calibration method was utilized for quantitation. Based on the chemical structure and chromatography performance, each analyte was attached to corresponding IL-ISs, which is recorded in Table S1. The volume of IL-ISs in the serial calibration solution was 12 μg/L. Matrix effects in the four cosmetic samples were determined by comparing the slopes of matrix-matched and solvent-only calibration curves, and the results show negligible effects following IL-IS calibration and MSPE purification. Therefore, solvent-only calibration curves can be directly adopted for quantitative detection. When the concentration of analytes in the acetonitrile extracts ranged from 0.2 to 20 μg/L, the calibration curve (the response and concentration ratio between analytes and ISs was ordinate and abscissa, respectively) displayed excellent linearity. The results are shown in Table S2. They indicate good linearity, with correlation coefficients (R^2^) ranging from 0.9984 to 0.9999. The LOD and LOQ were determined by spiking blank samples with standard solutions and identifying the concentrations that yielded signal-to-noise ratios of 3 and 10, respectively. The results show that the LOD and LOQ were 0.2~1.0 μg/kg and 1.0~3.0 μg/kg. The present method demonstrated great linearity and sensitivity, which rendered it suitable for measuring trace sex hormones in cosmetics.

Four kinds of blank matrices, including toner, lotion, gel, and cream, were prepared. Blank samples were added with three different spiked concentrations (4, 10, and 15 μg/kg) of analytes and 12 μg/kg of IL-ISs. The recovery was calculated by the calibration curve (the response ratio between analytes and ISs versus the concentration ratio between analytes and ISs). The results indicate that the recoveries of 16 analytes for four kinds of matrices were from 71.7% to 116.2%, with relative standard deviations (RSDs) ranging from 1.6% to 8.3% in Table S3. Moreover, the intra-day precisions were evaluated by analyzing six spiked parallel samples for four kinds of cosmetics in one day and the inter-day precisions for three consecutive days. As shown in Table S4, intra-day and inter-day precisions expressed by RSDs were 2.3–8.8% and 0.6–8.8%, respectively, proving the present method to be repeatable and accurate.

### 2.5. Method Comparison

The present work was compared with the published methods for detecting sex hormones in cosmetics, as summarized in Table 2. Existing research, as compiled in Table 2, falls into two main categories, HPLC and HPLC-MS, the latter of which offers higher detection sensitivity. Given the complex matrices of cosmetic samples, prior to LC separation, pretreatment techniques, including MSEBLLME [9], DES-MCG [26], MILs-DLLME [36], MISPE [37], and HILME [38], are generally required to minimize analytical interference. However, when using mass spectrometry for detection, complex pretreatment methods can be avoided [39]. The MEMMS mode used for data analysis in the literature [40] necessitates complex chemometrics operations and highly trained personnel. Consequently, that study only analyzed three sample batches, thereby limiting its practicality for high-throughput detection. The present method achieves a lower detection limit and broader analyte coverage (16 sex hormones) across diverse cosmetic types compared to existing mass spectrometry approaches. This performance is achieved by a simplified pretreatment that uses an external magnet for rapid phase separation, eliminating repeated centrifugation. This streamlines the workflow, reduces organic solvent consumption and energy use, and can be applied to the detection of large batches of samples. The magnetic microspheres used in this study are commercially available, ensuring mass producibility and high batch-to-batch stability [41,42]. Moreover, automated MSPE instruments are already available on the Chinese Market [43]. By applying the experimental parameters established herein, we are developing a fully automated protocol using such an instrument to achieve complete operational automation.

### 2.6. The Greenness of the Proposed Method

**Figure 3:**
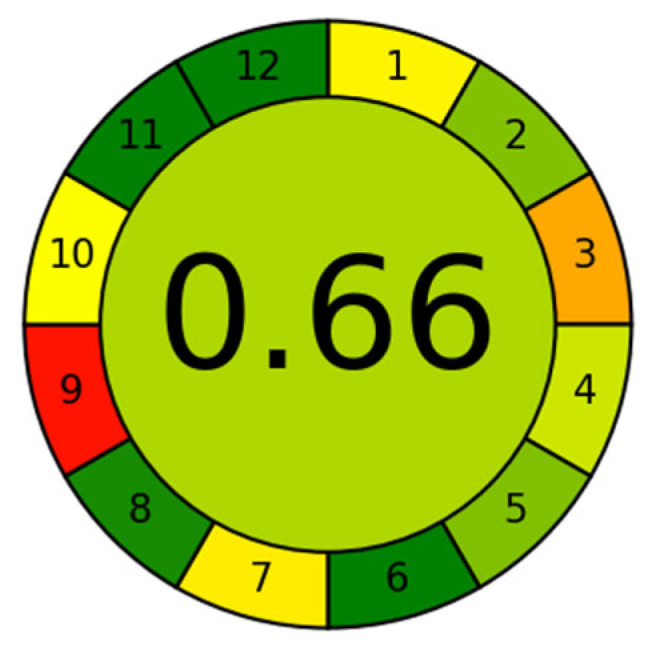
Application of the AGREE approach to evaluate the proposed method. 1. Sample treatment; 2. sample amount; 3. device positioning; 4. sample pretreatment stages; 5. automation and miniaturization; 6. derivatization; 7. waste; 8. analysis throughout; 9. energy consumption; 10. source of reagents; 11. toxicity; 12. operator’s safety.

An assessment of the method’s greenness was performed using the widely recognized Analytical Greenness Calculator (AGREE) [44,45]. Based on the 12 principles of green analytical chemistry, AGREE assigns a quantitative score (0–1) to analytical methods, visualized as a color-coded pictogram (red-yellow-green). A score exceeding 0.6 signifies acceptable greenness, while scores closer to 1 reflect better sustainability [46]. In this study, the developed method was evaluated using the Analytical Greenness Calculator (AGREE; https://mostwiedzy.pl/wojciech-wojnowski,174235-1/AGREE, accessed on 24 December 2025), yielding a score of 0.66 (Figure 3). This result demonstrates its good environmental friendliness. The proposed method offers several operational and safety advantages, such as streamlined processing, high analytical throughput, avoidance of centrifugation, and derivatization reagents. To further improve the method’s greenness, future work could focus on developing online sample pretreatment, using greener reagents, and minimizing solvent consumption.

### 2.7. Application 

To evaluate the feasibility of the current method, 116 batches of cosmetic samples were analyzed. These cosmetic samples involved moisturizers, skin-whitening and anti-acne products, hair care products, children’s cosmetics, and so on. The results are shown in Table 3. P was detected in 7 batches of hair loss prevention cosmetics and 1 batch of children’s cosmetics, ranging from 6.5 to 70.5 μg/kg. Meanwhile, 20α-DHP was found in 3 batches of hair loss prevention cosmetics, exhibiting a low detection concentration. Both E2 and E3 were detected in a single batch of children’s cosmetics at a low level, individually.

According to Regulation (EC) No. 1223/2009 of the European Parliament and the Council on Cosmetic Products (Annex III), the addition of pharmacologically active substances with estrogenic, androgenic, or gestagenic activity is prohibited in cosmetics. In China, the Cosmetics Safety Technical Specification issued by the National Medical Products Administration (NMPA) also explicitly lists substances with estrogenic, androgenic, or progestogenic effects as prohibited ingredients in cosmetics [47]. The trace levels of sex hormones measured in this study point to several possible sources, such as deliberate but undeclared addition, or their natural presence in raw material impurities. A wide variety of raw materials are used in cosmetics, including numerous plant-based ingredients [48,49]. Previous findings of P in some plant lend support to the latter [50], though further direct validation is required. It can also be inferred that contamination occurred during production or originated from the migration of chemical compounds from plastic material containers [51], although these have not been fully substantiated by the available data.

Abbreviations: MIN, Minoxidil; HYD, Hydrocortisone; DEX, Dexamethasone; MEM_MS_: Multiplicative effects model for mass spectroscopy; MILs-DLLME: magnetic ionic liquids–dispersive liquid–liquid microextraction; DES-MCG, deep eutectic solvent-based magnetic colloidal gel; MISPE, molecularly imprinted solid-phase extraction; MSEBLLME, Magnetically stirring extraction bar liquid–liquid microextraction; HILME, homogeneous ionic liquid microextraction; SPE, solid-phase extraction

### 2.8. Safety Evaluation 

Conducting a safety evaluation on potential hazardous substances in cosmetics can help people correctly understand the health risks they pose [52]. As can be seen from the above test results, P is the most frequently detected substance in hair loss prevention cosmetics. In addition, E2, E3, and 20α-DHP have also been detected. Due to the lack of relevant exposure data in cosmetics and the small sample size of the content data in cosmetic samples, it is hard to assess the safety of these three substances. Given that P is frequently detected and exhibits high biological activity, and the existing research data are relatively sufficient, this study focuses on the safety assessment of P. Hall et. al [53,54] provide the daily exposure levels and relevant data for different types of cosmetics involving bathing, showering, hair care, skin care, make-up, deodorant, and oral hygiene. Among them, the relative daily amount applied to shampoo is 150.49 mg/kg·bw/day, with a retention factor of 0.01, and the calculated relative daily exposure is 1.51 mg/kg·bw/day. According to the test results in Table 3, the content range of P in hair loss prevention cosmetics is 0.0065~0.036 mg/kg. Adopting the most conservative assessment method, the exposure of P in hair loss prevention cosmetics was calculated based on the concentration of 0.036 mg/kg. A 100% percutaneous absorption rate of P was applied as a conservative worst-case assumption. According to Formula (1), the SED of P in hair loss prevention cosmetics was determined to be 5.4 × 10^−6^ mg/kg·bw/day. 

Clinically, P is mainly administered via three routes: oral administration, intramuscular injection, and vaginal administration. Although P is also available as a topical skin ointment, detailed data on its percutaneous absorption and bioavailability remain insufficient. Based on available chronic toxicity data, NOAEL can be used to assess the dose–response relationship. In a 52-week study concerning repeated-dose toxicity of P administered via vaginal rings in rhesus monkeys [55], the maximum administered dose was reported to be 1770 μg/d. Based on a body weight of 2.5 kg, the corresponding NOAEL for P via vaginal administration was calculated to be 708 μg/kg·bw/d. Considering the high bioavailability of P and the similarity of percutaneous absorption [56,57], to assess the health hazards of long-term human exposure to P, the NOAEL value can be extrapolated to humans for calculating the MoS of percutaneous absorption of P from cosmetics. Using Formula (2), the MoS value for P in hair loss prevention cosmetics was calculated to be 1.3 × 10^5^, as shown in Table 4. According to the SCCS guidelines, an MoS greater than 100 indicates that the risk to human health from residual P in such products is relatively low. In addition to P, 20α-DHP, E2, and E3 were also detected in the hair loss prevention cosmetics and children’s cosmetics, respectively. Given the particular vulnerability of children’s skin and their critical stage of growth and development [58], conducting rigorous safety evaluations for sex hormones in children’s cosmetics is essential. 

## 3. Material and Methods

### 3.1. Chemicals and Reagents

The standards estriol (E3, 97.6%), estradiol (E2, 96.3%), ethynyl estradiol (EE2, 99.4%), gestrinone (GET, 99.7%), estrone (E1, 100%), methyltestosterone (MT, 99.5%), diethylstilbestrol (DES, 99.5%), norgestrel (NG, 99.4%), megestrol acetate (MGA, 99.2%), progesterone (P, 99.6%), chlormadinone acetate (CMA, 100%), and medroxyprogesterone acetate (MPG, 99.6%) were obtained from National Institutes for Food and Drug Control (Beijing, China). 20α-Hydroxyprogesterone (20α-DHP, 95.3%), 6-dehydroprogesterone (DelatP, 99.6%), estradiol-D5 (E2-D5, 99.0%), estrone-D2 (E1-D2, 99.2%), diethylstilbestrol-D8 (DES-D8, 98.5%), and progesterone-D9 (P-D9, 95.9%) were provided by CATO Research Chemicals Inc. (Guangzhou, China). Dienestrol (DIEP, 99.9%), hexoestrolum (HEX, 99.4%), and estriol-D3 (E3-D3) solutions in methanol (100 μg/mL) were purchased from Alta scientific (Tianjin, China). HPLC-MS grade methanol, acetonitrile, formic acid and ammonium formate were obtained from Thermo Fisher Scientific (MA, USA). Ammonium fluoride was supplied by Honeywell (Shanghai, China). The Fe_3_O_4_@HLB magnetic solid adsorbent (20–40 μm, 55–75 Å), Fe_3_O_4_@C18 (20–40 μm, 90–130 Å), and Fe_3_O_4_@C8 (20–40 μm, 90–130 Å) were provided by Pureton Lab Equipment (Shanghai, China). These magnetic solid adsorbents were dispersed in methanol at a concentration of 0.1 g/mL to take conveniently before use.

Stock standard solutions were prepared by dissolving solid analytes with methanol at a concentration level of 1000 μg/mL and stored at 4 °C in the dark. Each of the standard solutions was separately measured out, mixed with acetonitrile, and subjected to dilution to yield a working standard solution at a concentration of 10 μg/mL.

### 3.2. Apparatus

LC-MS/MS analysis was carried out using a 6495B Triple Quad MS detector (Agilent Technologies Inc., Santa Clara, CA, USA) with an Agilent Jet Stream Source interface coupled to a 1290 Infinity II chromatography system. The MassHunter software was used for system control and data acquisition. Scanning electron microscope (SEM) images were acquired by an S-4800 SEM (Hitachi, Tokyo, Japan). Other instruments, an electronic balance, an ultrasonic device, a vortex mixer, and a centrifuge (Beili Medical, Beijing, China), were employed for the sample pretreatment.

### 3.3. Sample Preparation

A total of 116 kinds of cosmetic samples were available at and purchased from online e-shops and local markets. Sample types mainly include toner, lotion, gel, and cream and are used for moisturizing, skin whitening, acne prevention, and hair loss prevention.

The cosmetic sample (0.5 g) was put into a 15 mL polypropylene centrifuge tube, and the IL-ISs were added to the samples before sample pretreatment. Firstly, it was extracted with 10 mL of acetonitrile, followed by vortex for 30 s, sonicated for 5 min, and centrifuged for 5 min at 6533× *g*. Then, 5 mL of supernatant was transferred in a 50 mL polypropylene centrifuge tube, mixed with 45 mL of ultra-pure water, and subsequently mixed with 30 mg of Fe_3_O_4_@HLB adsorbents. The mixtures were shaken for 5 min at 750× *g* as the adsorption process. The Fe_3_O_4_@HLB adsorbents with analytes and supernatant were separated through an external magnet field (as illustrated in Figure S6). After decanting the supernatant, concentrated sex hormones were desorbed by 0.5 mL of acetonitrile after shaking for 5 min at 750× *g*. Finally, the eluent was injected into the UHPLC-MS/MS for analysis.

### 3.4. LC-MS/MS Instrument Parameters

The LC-MS/MS conditions were as detailed in our previous report [59]. In this experiment, separation was achieved on a Waters ACQUITY UPLC BEH C18 column (1.7 μm, 2.1 × 150 mm). The mobile phase consisted of 0.1 mM of an ammonium fluoride aqueous solution (A) and acetonitrile (B), with a flow rate of 0.3 mL/min. The gradient elution procedure was set as follows: 0–2 min, 10–50% B; 2–7 min, 50–60% B; 7–8 min, 60–75% B; 8–9 min, 75–80% B; 9–11 min, 80–95% B; 11–14 min, maintained at 95% B; 14–14.1 min, 95–10% B; postrun 2 min. The column temperature was kept at 30 °C and the injection volume was 10 μL. The instrument source parameters were set as follows: gas temperature, 250 °C; gas flow, 115 L/min; nebulizer, 35 psi; sheath gas temperature, 350 °C; sheath gas flow, 12 L/min; capillary voltage, 3500 V (positive mode) and 2500 V (negative mode); and nozzle voltage, 1500 V (positive mode) and 2000 V (negative mode).

### 3.5. Safety Evaluation of P in Cosmetics

A preliminary safety evaluation was conducted based on the detected result to identify potential health risks posed by the residual sex hormones. In 2023, the Scientific Committee on Consumer Safety (SCCS) published the guidelines “the SCCS notes of guidance for the testing of cosmetic ingredients and their safety evaluation (12th revision) (SCCS/1647/22)” [60]. The assessment process was carried out in accordance with these guidelines. The systemic exposure dose (SED, mg/kg·bw/day) was calculated by Formula (1):

SED = E_product_ × C × DAp (1)

where E_product_ (mg/kg·bw/day) is the estimated daily exposure to a cosmetic product per kg body weight, based upon the amount applied and the frequency of application; C (%) is the concentration of the substance under study in the finished cosmetic product on the application site; and DAp (%) represents dermal absorption. In the case of a threshold effect, the margin of safety (MoS), which was dimensionless, was calculated by Formula (2):

MoS = NOAEL/SED(2)

where no observed adverse effect level (NOAEL) is defined as the highest dose or exposure level where no (adverse) treatment-related findings are observed. For cosmetic ingredients, NOAEL is mainly derived from a repeated-dose animal study or from a reproductive-toxicity animal study.

## 4. Conclusions

In this research, a new approach using MSPE sample pretreatment and the IS calibration method was optimized and validated for the determination of 16 kinds of sex hormones in cosmetics by UHPLC-MS/MS. This proposed sample pretreatment makes it possible to achieve the extraction of sex hormones, sample purification, and enrichment. The IL-ISs were added into the cosmetics before pretreatment, and internal standard calibration was employed. Furthermore, it made great progress in the detection sensitivity of trace analytes, achieving an AGREE score of 0.66 (out of 1), confirming its environmental friendliness. Finally, 116 batches of real cosmetic samples were analyzed using the current method, and 10 samples were found to contain trace amounts of sex hormones. A preliminary safety evaluation by calculating the margin of safety (MoS) for P in hair loss prevention cosmetics revealed low health risks. On the whole, the proposed method possesses potential to provide new angles and insights for detecting trace sex hormones in cosmetics.

## Figures and Tables

**Table 1:** General information and optimized mass parameters for 16 target compounds and the corresponding isotope-labeled internal standards.

No.	Abbreviation	Precursor Ion (*m*/*z*)	Product Ion (*m*/*z*)	CE (V)	RT (min)	Detect Mode
1	E3	287.1	171.0 *, 145.0	43, 43	3.4	ESI-
2	E3-D3	289.7	173.0 *, 146.8	42, 48	3.4	ESI-
3	E2	271.2	183.3 *, 145.2	49, 47	4.8	ESI-
4	E2-D5	276.3	187.2 *, 147.2	49, 47	4.8	ESI-
5	EE2	295.2	159.0 *, 145.0	43, 47	5.3	ESI-
6	GET	309.1	241.1 *, 199.2	30, 44	5.5	ESI+
7	MT	303.2	97.0 *, 108.9	30, 29	5.6	ESI+
8	E1	269.1	145.1 *, 143.1	43, 61	5.6	ESI-
9	E1-D2	271.2	147.1 *, 145.1	37, 69	5.6	ESI-
10	DES	267.1	222.2 *, 251.2	42, 35	5.9	ESI-
11	DES-D8	275.2	259.2 *, 228.1	28, 38	5.9	ESI-
12	HEX	268.8	119.1 *, 134.0	41, 15	6.2	ESI-
13	DIEP	264.9	93.1 *, 259.0	32, 26	6.2	ESI-
14	NG	313.2	109.0 *, 245.3	33, 24	6.4	ESI+
15	20α-DHP	317.3	97.1 *, 109.0	29, 29	6.8	ESI+
16	DelatP	313.2	43.1 *, 159.1	73, 25	7.7	ESI+
17	MGA	385.1	267.3 *, 325.1	24, 20	8.3	ESI+
18	P	315.2	109.0 *, 97.3	30, 30	8.5	ESI+
19	P-D9	324.4	100.2 *, 112.8	25, 25	8.3	ESI+
20	CMA	405.1	309.3 *, 267.3	23, 30	8.7	ESI+
21	MPG	387.2	122.9 *, 327.0	35, 18	8.8	ESI+

* represents quantitative ion.

**Table 2:** Comparison with previous methods referring to the determination of sex hormones in cosmetics.

No.	Cosmetics	Analysis Throughout	Analytes	Pretreatment Process	Pretreatment Time (min)	Analytical Methods	LOD	LOQ	Reference
1	-	8	Nandrolone, testosterone, E1, 17a-DHP, MPG, MGA, P, and androlin	Dilution, pH adjustment, and MSEBLLME	>60	HPLC-DAD	0.91–1.01 ng/mL	3.04–5.97 ng/mL	[9]
2	Toner	4	EE, NG, MA, and MPA	Dried under nitrogen, redissolved, and DES-MCG	<60	HPLC-DAD	1.2~6.6 ng/mL	4.4~26.6 ng/mL	[26]
3	Lotion	5	E1, E2, CMA, MGA, and MPA	Centrifugation and MILs-DLLME	>15	HPLC-UV	5~15 ng/mL	15~35 ng/mL	[36]
4	Lotion, emulsion, and cream	6	P, E1, E3, DES, DEX, and HYD	Centrifuged, dried under nitrogen, redissolved, and MISPE	- ^a^	HPLC-UV	16 ng/mL	50 ng/mL	[37]
5	Liquid cosmetics and gel-like cosmetics	8	P, 17α-E3, 17α-ethinylestradiol, E1, 17α-DHP, MPG, MGA, and norethisterone acetate	Dilution, pH adjustment, HILME, and centrifugation	-	HPLC-DAD	0.03~0.24 ng/mL	0.1~0.78 ng/mL	[38]
6	Toner and Lotion	2	E1 and P	Solvent extracted, centrifugation, and filtration	41.5	HPLC-MS/MS	<25 μg/kg	100 μg/kg	[17]
7	Toner and lotion	4	MT, CMA, MPG, and P	Solvent extracted, filtration, and SPE	>20	HPLC-Q-TOF-HRMS	10.43~12.43 μg/kg	11.89~22.59 μg/kg	[39]
8	Cleaning toner and essence emulsion	4	E1, P, MIN, and HYD	Ultrasonic bath and refrigerated centrifugation	>35	HPLC-MS/MS with MEM_MS_ model	0.03~1.0 μg/kg	0.07~3.0 μg/kg	[40]
9	Toner, lotion, gel, and cream	16	E1, E2, E3, EE2, GET, MT, DES, HEX, DIEP, NG, 20α-DHP, DelatP, MGA, P, CMA, and MPG	Solvent extracted and MSPE	20.5	UHPLC-MS/MS	0.2~1.0 μg/kg	1.0~3.0 μg/kg	The present method

^a^ not clearly stated.

**Table 3:** Determination results of sex hormones in real cosmetics by the present method ^a^.

No.	Cosmetic Category	Detected		Detected	
		Compound	Amount (μg/kg)	Compound	Amount (μg/kg)
1	hair loss prevention	P	20.6	20α-DHP	7.6
2	hair loss prevention	P	27.2	20α-DHP	7.5
3	hair loss prevention	P	36.3	20α-DHP	7.9
4	hair loss prevention	P	22.0	/	/
5	hair loss prevention	P	30.4	/	/
6	hair loss prevention	P	6.5	/	/
7	hair loss prevention	P	7.7	/	/
8	children’s cosmetics	P	70.5	/	/
9	children’s cosmetics	E3	13.1	/	/
10	children’s cosmetics	E2	13.7	/	/

^a^ Only positive samples are shown.

**Table 4:** Safety evaluation progesterone exposure in hair loss prevention cosmetics.

Cosmetic Product Categories	Detected Results (mg/kg)		Daily Exposure Level ^a^					SED ^b^ (μg/kg·bw/day)	NOAEL ^c^ (μg/kg·bw/d)	MoS ^d^
	Mean + SD	Maximum	Estimated Daily Amount Applied (g/d)	Relative Daily Amount Applied	Retention Factor	Calculated Daily Exposure E_product_ (g/d)	Calculated Relative Daily Exposure E_product_/bw (mg/kg·bw/day)			
Hair loss prevention	0.022 ± 0.011	0.0363	10.46	150.49	0.01	0.11	1.51	5.4 × 10^−3^	708	1.3 × 10^5^

^a^ The data used are those of hair care cosmetics sourced from SCCS/1647/22; ^b^ calculated via Equation (1), where the percutaneous absorption rate is set at 100%, and the content in the sample was calculated based on the maximum detected value; ^c^ the data are from reference [57]; ^d^ calculated by Formula (2) based on the values of SED and NOAEL.

## Data Availability

Data will be made available on request.

## Supplementary Materials

The following supporting information can be downloaded at: https://www.mdpi.com/article/10.3390/molecules31010090/s1, Figure S1: Scanning electron microscope images (SEM) of Fe_3_O_4_@ HLB magnetic material; Figure S2: Effects of different concentrations of ammonium fluoride on relative intensity for sex hormones; Figure S3: Effects of different column temperature on relative intensity for sex hormones; Figure S4: Effect of types of extraction solvent on extraction efficiency of sex hormones in 4 kinds of cosmetics (A: Toner; B: Lotion; C: Gel; D: Cream); Figure S5: Effect of sonication time on extraction efficiency of sex hormones in 4 kinds of cosmetics (A: Toner; B: Lotion; C: Gel; D: Cream); Figure S6: The photos of magnetic beads in the solution achieving phase separation with the assistance of the magnetic rack. The commercial magnetic rack (A), and the front view (B), the top view (C) of the magnetic rack when loaded with two 50 mL centrifuge tubes, as well as its state after the centrifuge tubes are removed from the rack (D).; Table S1: Correspondence between analytes and their isotope-labelled internal standards; Table S2: The linear ranges, determination coefficient (R^2^), limit of detection (LOD) and limit of quantitation (LOQ) of 16 kinds of sex hormones; Table S3: Average recoveries and relative standard deviations (RSDs) (n = 6) of the 16 kinds of sex hormones in different cosmetics (%); Table S4: The intra-day (n = 6) and inter-day (n = 3) RSDs of peak area of the present method (%).
