# Supplementary material for: Targeted Determination of Residual Sex Hormones in Cosmetics Using Magnetic Solid-Phase Extraction with Isotope-Labeled Internal Standards by UHPLC-MS/MS
Source: Molecules. 2025 Dec 25;31(1):90. doi: 10.3390/molecules31010090 (PMC12787144; doi:10.3390/molecules31010090)
Supplement: Supplementary file 1 [file molecules-31-00090-s001.zip › molecules-4029911-supplementary.pdf]

molecules

Electronic Supplementary Information(ESI)

Targeted Determination of Residual Sex Hormones in Cosmetics Using Magnetic Solid-Phase Extraction with Isotope-Labeled Internal Standards by UHPLC-MS/MS

Yalei Dong[1,†], Shuyan Sun[1,2,†], Yasen Qiao[1], Chunhui Yu[3], Haiyan Wang[1,*], and Lei Sun[1,*]

[1] National Institutes for Food and Drug Control/NMPA Key Laboratory for Research and Evaluation of Cosmetics, Beijing 100050, China; dongyalei@nifdc.org.cn (Y.D.); yan18531864610@163.com (S.S.); qiaoyasen@126.com (Y.Q.)
[2] School of Traditional Chinese Pharmacy, NMPA Key Laboratory for Research and Evaluation for Cosmetics, China Pharmaceutical University, Nanjing 211198, China
[3] Pureton Lab Equipment (Shanghai) Co., Ltd., Shanghai 201108, China; 13825165882@139.com
Correspondence: Correspondences. Tel: +86-010-67095060. E-mail address: Summerwhy163@163.com (H. W.), dasunlei@sina.com (L. S.).
[†] These authors contribute equally to this work.

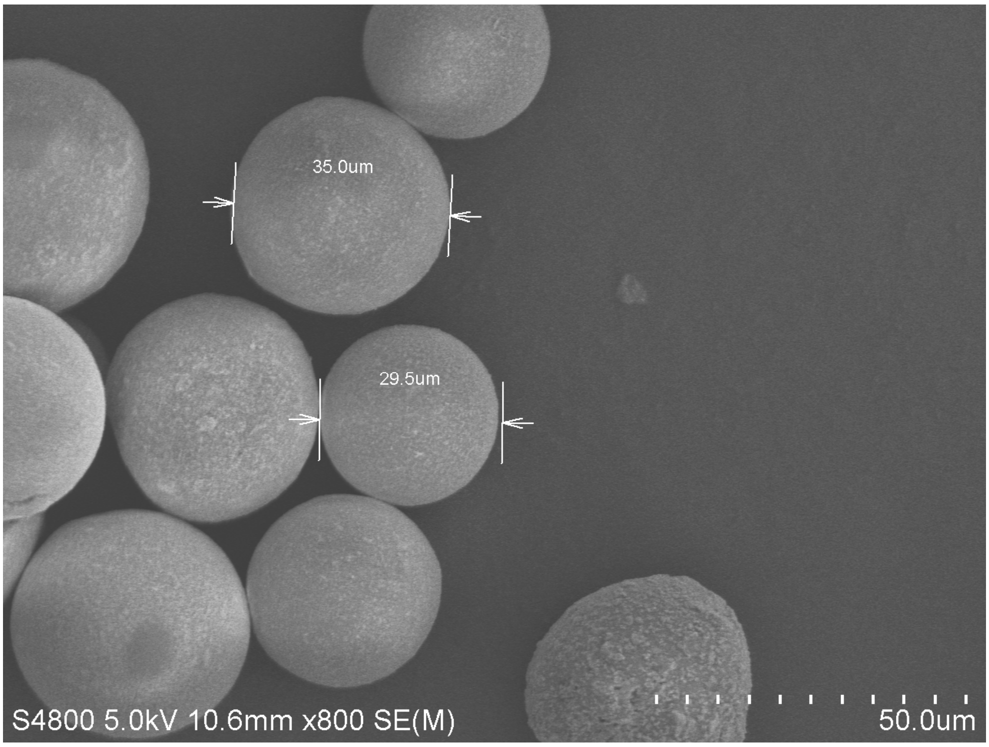

Figure S1. Scanning electron microscope images (SEM) of Fe_3O_4@ HLB magnetic material

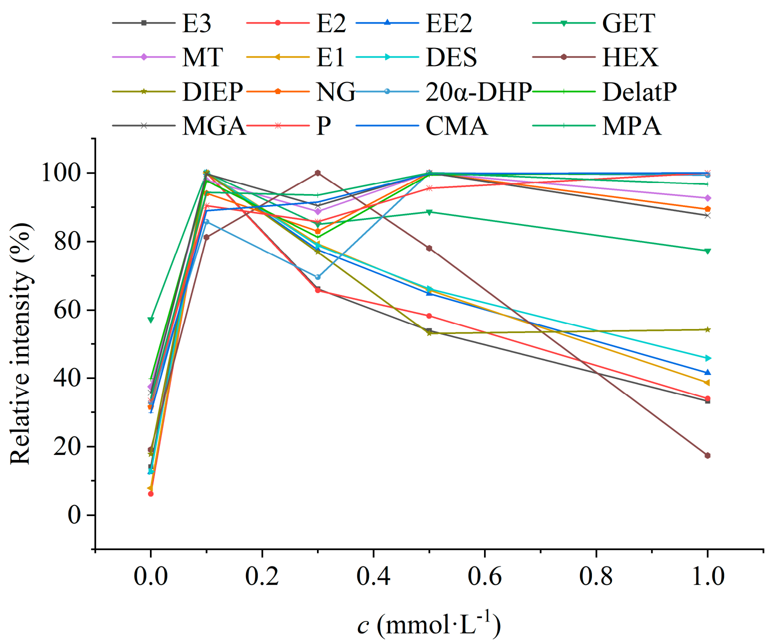

Figure S2. Effects of different concentrations of ammonium fluoride on relative intensity for sex hormones

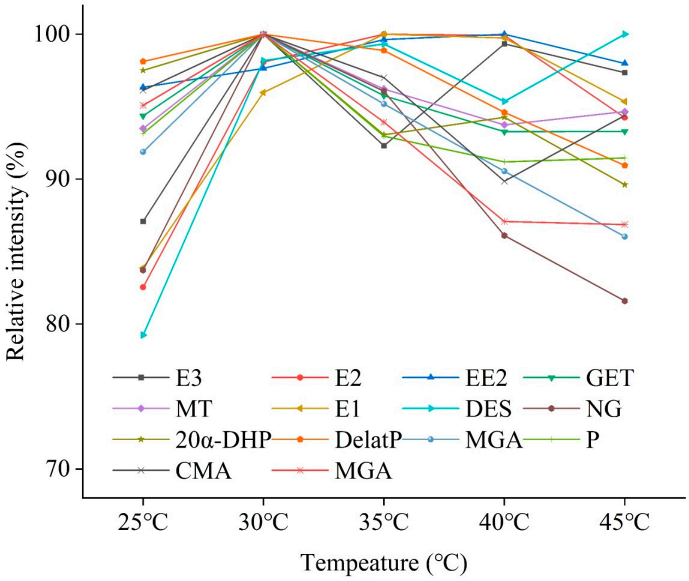

Figure S3. Effects of different column temperature on relative intensity for sex hormones

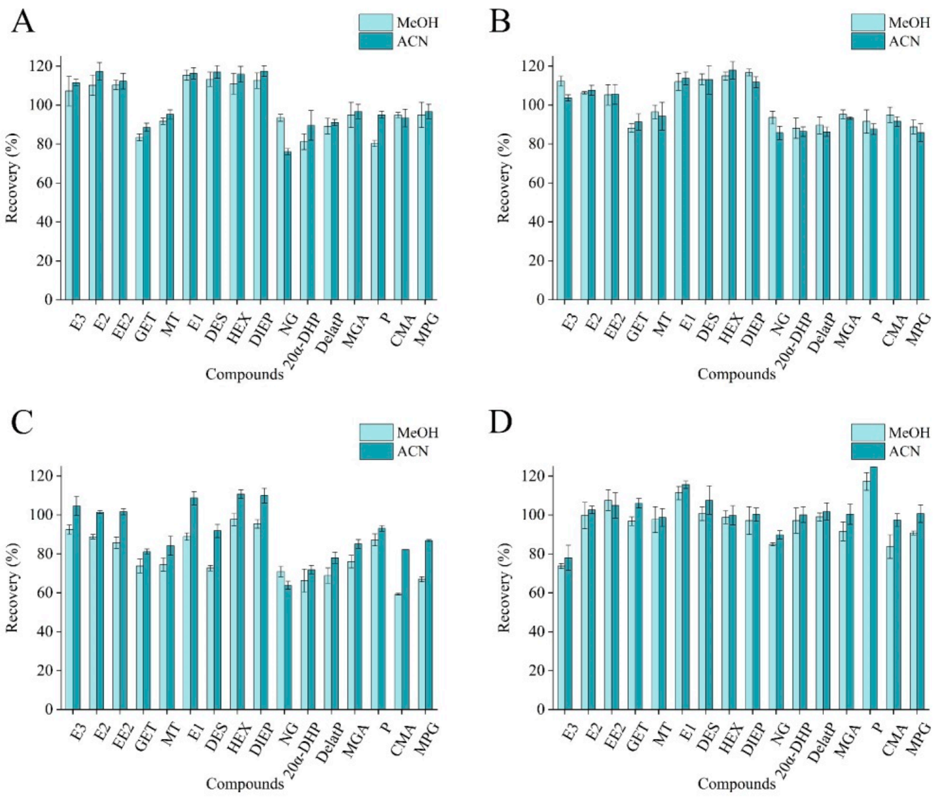

Figure S4. Effect of types of extraction solvent on extraction efficiency of sex hormones in 4 kinds of cosmetics (A: Toner; B: Lotion; C: Gel; D: Cream)

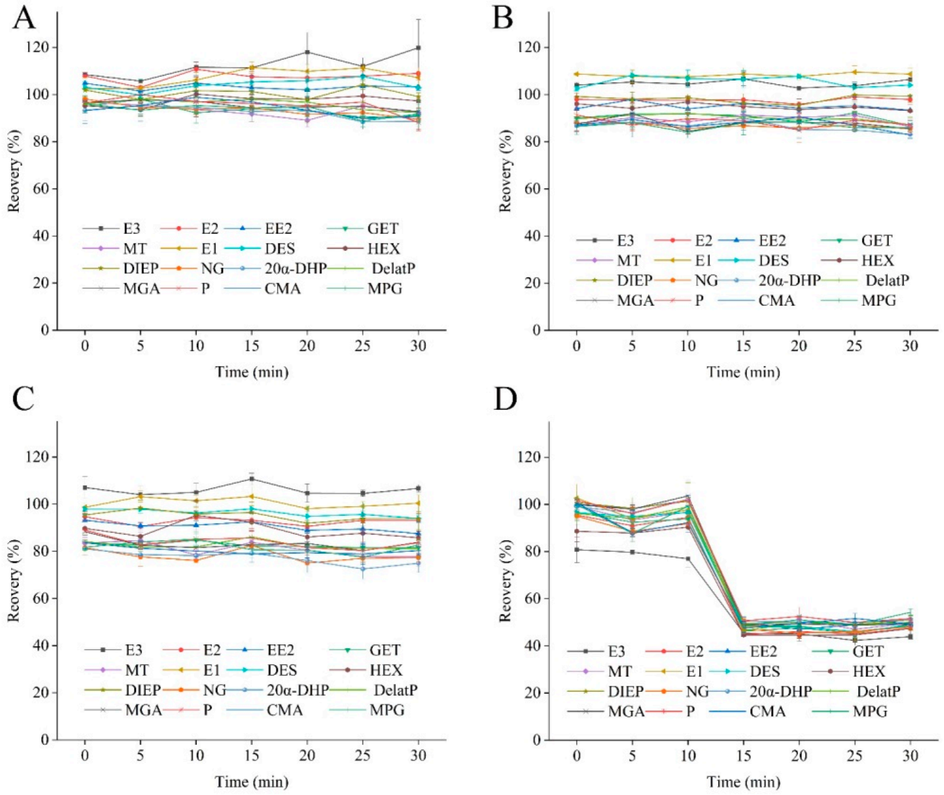

Figure S5. Effect of sonication time on extraction efficiency of sex hormones in 4 kinds of cosmetics (A: Toner; B: Lotion; C: Gel; D: Cream)

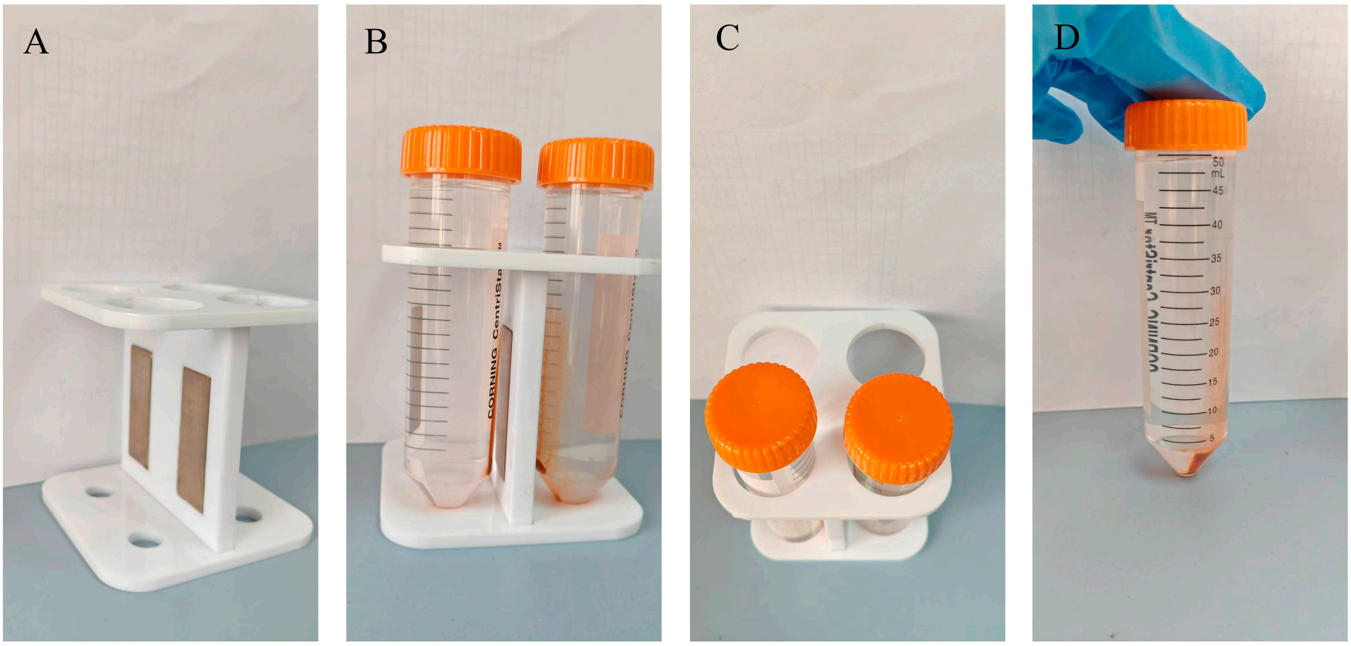

Figure S6. The photos of magnetic beads in the solution achieving phase separation with the assistance of the magnetic rack.

The commercial magnetic rack (A), and the front view (B), the top view (C) of the magnetic rack when loaded with two 50 mL centrifuge tubes, as well as its state after the centrifuge tubes are removed from the rack (D).

Table S1 Correspondence between analytes and their isotope-labelled internal standards

**Isotope-labelled internal standards**	**Analytes**
E3-D3	E3
E2-D5	E2
E1-D2	E1
	EE2
DES-D8	DES
	HEX
	DIEP
P-D9	GET
	MT
	NG
	20α-DHP
	DelatP
	MGA
	P
	CMA
	MPG

Table S2 The linear ranges, determination coefficient (R^2), limit of detection (LOD) and limit of quantitation (LOQ) of 16 kinds of sex hormones

Compound Abbreviation	Linear range (μg/L)	Determination coefficient (R^2)	LOD (μg/kg)	LOQ (μg/kg)
E3	0.2 - 20	0.9985	0.6	2.0
E2	0.2 - 20	0.9991	0.6	2.0
EE2	0.2 - 20	0.9995	0.6	2.0
GET	0.2 - 20	0.9995	0.2	1.0
MT	0.2 - 20	0.9996	0.2	1.0
E1	0.2 - 20	0.9999	0.2	1.0
DES	0.2 - 20	0.9999	0.2	1.0
HEX	0.2 - 20	0.9999	0.2	1.0
DIEP	0.2 - 20	0.9995	0.2	1.0
NG	0.2 - 20	0.9990	0.6	2.0
20α-DHP	0.2 - 20	0.9984	0.6	2.0
DelatP	0.2 - 20	0.9992	1.0	3.0
MGA	0.2 - 20	0.9996	0.2	1.0
P	0.2 - 20	0.9998	0.2	1.0
CMA	0.2 - 20	0.9998	0.6	2.0
MPG	0.2 - 20	0.9999	0.6	2.0

Table S3 Average recoveries and relative standard deviations (RSDs) (n=6) of the 16 kinds of sex hormones in different cosmetics (%)

Compound Abbreviation	Added (μg/kg)	Toner		Lotion		Gel		Cream	
		Recovery	RSD	Recovery	RSD	Recovery	RSD	Recovery	RSD
	4.1	102.8	5.1	100.0	3.0	95.2	3.0	104.6	4.4
E3	10.2	108.3	2.7	106.1	2.7	103.3	5.6	103.5	5.6
	15.2	105.5	4.0	103.1	4.2	102.5	4.3	101.6	4.6
	4.0	95.1	6.0	95.6	3.6	97.9	6.2	94.7	4.5
E2	10.0	104.8	7.2	94.5	6.5	105.8	5.4	107.7	3.9
	15.0	102.3	3.8	104.1	5.2	98.9	3.9	101.5	5.0
	4.1	92.5	4.5	106.4	5.5	102.1	7.5	102.9	5.7
EE2	10.3	86.8	5.5	108.5	4.5	112.9	4.1	110.0	3.7
	15.4	83.8	6.6	100.7	3.2	105.9	3.4	101.7	2.8
	4.1	80.1	3.8	75.4	4.7	72.7	3.6	75.4	6.3
GET	10.2	82.7	4.2	73.6	7.1	76.5	3.5	73.6	2.3
	15.2	84.7	7.0	74.5	3.1	75.8	4.4	72.3	5.7
	4.1	88.6	6.6	91.1	6.1	83.2	7.6	76.2	2.2
MT	10.2	87.3	7.0	104.0	5.3	94.1	4.8	74.7	6.4
	15.4	99.1	7.8	102.7	7.1	95.9	5.6	75.6	2.4
	4.2	97.0	3.6	100.0	3.2	93.2	5.1	95.9	6.5
E1	10.5	104.2	4.6	107.0	5.3	107.8	2.8	104.8	6.2
	15.7	102.8	7.4	107.8	2.1	111.5	3.4	102.4	3.9
	4.1	94.1	6.3	100.5	4.4	90.6	5.0	97.2	6.1
DES	10.2	99.5	4.5	98.0	2.9	101.4	4.8	107.8	2.6
	15.3	104.3	5.9	98.0	5.0	99.7	6.2	101.8	3.9
	4.0	97.9	4.8	113.3	2.6	113.9	3.8	105.0	3.9
HEX	10.0	105.6	4.1	111.0	3.0	113.6	4.0	109.9	5.4
	15.0	102.8	4.1	105.5	2.9	112.2	4.1	105.6	3.2
	4.0	104.8	4.6	113.9	3.7	114.3	3.1	107.6	4.0
DIEP	10.0	114.0	3.4	111.0	2.8	111.4	3.3	113.6	2.8
	15.0	111.8	2.1	113.0	2.5	106.0	3.9	108.9	3.6
	4.0	106.0	6.1	101.4	5.3	97.8	6.6	86.1	5.2
NG	10.0	109.1	5.3	104.3	6.7	98.2	6.7	89.5	4.2
	15.0	105.7	4.1	99.6	4.5	99.5	4.4	83.8	2.0
	4.0	115.3	1.8	113.3	3.9	113.0	3.2	101.6	4.8
20α-DHP	10.1	116.2	2.3	111.6	4.5	114.9	4.3	102.8	6.9
	15.1	114.7	3.7	107.8	3.2	110.7	3.1	92.9	1.6
	4.1	113.5	4.1	109.1	5.8	104.9	7.3	73.7	8.3
DelatP	10.1	115.6	3.2	113.4	4.8	110.1	6.8	72.1	2.9
	15.2	115.6	3.8	108.5	5.0	109.7	4.9	77.3	6.2
	4.1	88.6	5.2	88.6	5.2	76.2	5.0	83.1	5.4
MGA	10.2	91.0	4.6	91.0	4.6	84.7	7.2	87.8	7.9
	15.3	85.3	5.0	85.3	5.0	81.2	4.5	93.5	6.5
	4.1	104.7	3.1	98.1	5.0	92.8	2.7	103.2	7.6
P	10.3	110.8	3.2	102.2	4.7	100.4	7.6	105.7	6.0
	15.5	106.2	4.4	103.4	4.0	99.4	5.8	97.3	6.7
	4.3	72.7	2.1	73.4	4.3	71.9	5.4	77.1	4.7
CMA	10.8	73.2	6.0	71.8	3.6	71.7	2.6	72.9	3.6
	16.1	75.1	5.7	74.1	5.1	77.7	5.9	73.8	3.9
	4.2	80.2	6.0	79.7	6.3	72.4	5.9	84.0	6.0
MPG	10.5	80.7	6.6	79.1	7.8	73.0	3.2	90.4	3.2
	15.7	79.8	2.1	74.9	3.8	77.3	5.7	90.0	4.1

Table S4 The intra-day (n=6) and inter-day (n=3) RSDs of peak area of the present method (%)

Compound Abbreviation	Toner		Lotion		Gel		Cream	
	Intra-day	Inter-day	Intra-day	Inter-day	Intra-day	Inter-day	Intra-day	Inter-day
E3	3.3	1.3	5.8	1.2	3.1	0.7	5.0	4.1
E2	6.4	5.5	6.5	7.9	6.9	2.0	5.6	1.4
EE2	5.0	5.9	7.2	0.9	4.0	5.2	5.8	5.9
GET	8.8	7.3	4.0	1.1	2.3	4.5	3.7	3.1
MT	6.2	8.0	6.2	3.1	7.4	4.8	7.0	5.2
E1	3.9	2.4	4.3	3.4	3.6	3.7	6.1	2.3
DES	6.2	2.7	4.2	1.0	4.2	2.0	3.9	4.1
HEX	4.8	6.3	5.2	1.8	4.5	2.5	7.1	8.8
DIEP	6.7	3.8	3.2	2.1	6.9	3.1	6.2	6.8
NG	6.6	5.0	5.3	2.6	4.7	5.0	6.7	4.0
20α-DHP	4.2	5.3	4.0	3.6	4.2	1.7	6.9	8.8
DelatP	6.9	5.3	2.6	1.5	5.7	3.6	8.4	8.2
MGA	3.8	2.0	3.6	3.6	4.9	3.4	5.4	0.9
P	5.3	0.8	3.6	1.0	8.1	4.1	6.8	3.6
CMA	5.0	4.5	4.7	7.4	6.8	8.1	4.7	0.6
MPG	7.7	6.2	6.2	8.4	7.9	8.3	8.3	1.8
